# The Effect of Proton Pump Inhibitor Use on Renal Function in Kidney Transplanted Patients

**DOI:** 10.3390/jcm9010258

**Published:** 2020-01-18

**Authors:** Dominik J. G. Flothow, Barbara Suwelack, Hermann Pavenstädt, Katharina Schütte-Nütgen, Stefan Reuter

**Affiliations:** Department of Medicine D, University Hospital of Muenster, 48149 Münster, Germany; d_flot01@uni-muenster.de (D.J.G.F.); barbara.suwelack@ukmuenster.de (B.S.); hermann.pavenstaedt@ukmuenster.de (H.P.); Katharina.schuette-nuetgen@ukmuenster.de (K.S.-N.)

**Keywords:** proton pump inhibitor, kidney transplantation, transplant rejection, GFR

## Abstract

Recently, proton pump inhibitor (PPI) intake has been linked to acute kidney injury and chronic kidney disease. The objective of this study was to assess the effect of PPIs on renal function and rejection rate in kidney transplant patients. We performed a single center, retrospective analysis of 455 patients who received a kidney transplant between May 2010 and July 2015. Median follow-up time was 3.3 years. PPI prescription was assessed in half-year intervals. Primary outcome parameters were the estimated glomerular filtration rate (eGFR), change in the eGFR, and >30% and >50% eGFR decline for different time periods (up to four years post-transplantation). Our secondary outcome parameter was occurrence of biopsy proven acute rejection (BPAR) in the first two years after transplantation. Except for >30% eGFR decline from half a year to two years post-transplantation (*p* = 0.044) and change in the eGFR, >30% and >50% eGFR decline showed no association with PPI intake in our patient cohort (*p* > 0.05). Similarly, by analyzing 158 rejection episodes, BPAR showed no correspondence with mean daily PPI intake. We conclude that prolonged PPI intake has no relevant adverse effect on kidney transplant function or rejection rates. Polypharmacy, however, remains a problem in renal transplant recipients and it is thus advisable to question the necessity of PPI prescriptions when clear indications are missing.

## 1. Introduction

With only a single transplanted kidney and oftentimes reduced renal filtration rates, kidney transplant (KTx) recipients are particularly vulnerable to the nephrotoxic adverse effects of drugs. Care is taken to avoid such drugs that could further impair kidney function. For this reason, recent epidemiological studies that have observed a relationship between acute kidney injury (AKI), chronic kidney disease (CKD) and proton pump inhibitor (PPI) intake have been of special interest for practitioners involved in the care of KTx patients [1,2,3,4,5].

Furthermore, in the setting of KTx, two medication interactions of possible relevance are the interaction between PPIs and mycophenolate mofetil (MMF) and between PPIs and tacrolimus. The first may lead to decreased blood levels of the active metabolites of MMF [6,7,8,9,10,11,12], which may result in increased rejection rates [13,14,15,16]. Tacrolimus is known to be nephrotoxic and it is thought that interactions with PPIs may change its uptake and/or metabolism [17,18,19], potentially increasing tacrolimus blood concentration. This could be detrimental to kidney transplant function.

In the face of surgical stress and long-term polypharmacy, upper gastrointestinal symptoms are frequent in KTx patients [20]. Even in those patients receiving acid suppressive therapy, the risk of ulcer disease is still elevated [20]. Gastrointestinal complications have also been associated with decreased graft survival [21]. As PPIs are a very effective form of acid suppression, they are frequently given as prophylaxes among transplant recipients [20,22,23]. In our center, they are the standard of care for KTx patients.

In light of the mentioned studies and the possibility of adverse drug interactions between PPIs and mycophenolate mofetil or tacrolimus, we carried out this study. Previous PPI studies in KTx patients have focused on the possible interaction of MMF with PPIs [22,24,25] or the occurrence of other PPI intake related adverse events [23]. We retrospectively evaluated if a relationship between PPI intake and renal function could be found. We analyzed follow-up data of up to four years after transplantation. To our knowledge, no study exists to date that has specifically analyzed the changes in renal function after KTx with regard to PPI intake over a comparable time frame. Additionally, we compared rejection rates, as these may be of relevance regarding MMF and PPI interactions.

## 2. Patients and Methods

### 2.1. Patients

Prior to analysis, the data of all patients was anonymized. The local ethics committee (Ethik Kommission der Ärztekammer Westfalen-Lippe und der Medizinischen Fakultät der Westfälischen Wilhelms-Universität, No. 2014-381-f-N) approved the study. Methods in this study were carried out in accordance with the current transplantation guidelines and the Declarations of Istanbul and Helsinki. Written informed consent was given by all participants at the time of transplantation for recording their clinical data.

We herein performed an explorative, retrospective, single-center cohort study. We enrolled all patients receiving a KTx between May 2010 and July 2015 at the University Hospital Muenster. Inclusion criteria were patient age ≥18 years and PPI therapy at primary hospital discharge post-transplantation (pTx). Patients receiving multiple organ transplants remained included. The recipient and donor data was collected from the patients’ electronic files. The following data was collected and used: recipient and donor age and sex; recipient body mass index (BMI); prior renal transplants; transplant under European Senior Program; donor type (living or deceased); delayed graft function (DGF; dialysis within the first week pTx); cold ischemia time; pre-transplant time dialyzed; pre-transplant arterial hypertension; pre-transplant diabetes; presence of peripheral arterial occlusive disease; cerebral arterial occlusive disease or stroke; coronary heart disease or myocardial infarction; anticoagulant prescription; antiplatelet drug prescription (including acetylsalicylic acid (ASA)); statin prescription; MMF dosage at primary discharge and two years pTx; cortisone intake at primary discharge and one year pTx; tacrolimus dose and blood level three months pTx; prior smoking history; continuation of smoking after KTx; number of human leukocyte antigen (HLA) mismatches; ABO incompatibility of transplant; induction therapy; pre-transplant donor specific antibody (DSA) occurrence; panel reactive antibodies; transplant rejection occurrence and type according to Banff criteria; eGFR from half a year to four years pTx; and data on PPI prescription. At primary discharge, none of the patients had any non-steroidal anti-inflammatory drug prescriptions, except for ASA. To improve adjustment for confounding through comorbidities, additional data was collected to calculate the Charlson comorbidity index [26] at transplantation [27].

The induction therapy was chosen according to the immunological risks of the patients. One gram of mycophenolate mofetil was given twice a day; the dosage was reduced in case of adverse events. Prednisolone was started at 500 mg intravenously (i.v.) before KTx, followed by 100 mg for three days; then reduced by 20 mg/day. A dosage of 20 mg/day was maintained until day 30 and then slowly reduced to 5 mg/day. Immunosuppressive maintenance therapy usually consisted of a calcineurin inhibitor (tacrolimus or cyclosporine A), mycophenolate sodium or mycophenolate mofetil and prednisolone.

### 2.2. Proton Pump Inhibitors—Data Collection

Data on the prescription of PPIs (agent and dose prescribed) were collected in half year intervals for all patients starting at primary discharge. PPI intake was assumed according to this prescription until the next interval. If data was not present for a certain interval, the prescription from the preceding interval was assumed.

Due to the favorable drug interaction profile of pantoprazole in comparison to other PPIs [28], pantoprazole is used as the PPI of choice at our center. Therefore, it has also been used as the standard PPI in this study. In order to also use data from different PPIs, equivalent doses were calculated for the two other agents (omeprazole and esomeprazole) used. We used the ratio: 40 mg pantoprazole ≙ 20 mg omeprazole ≙ 20 mg esomeprazole [29,30].

At our center, patients are instructed to ingest tacrolimus and MMF on an empty stomach one hour before intake of PPIs. Both tacrolimus and MMF are usually taken twice daily, while PPIs are mostly taken once per day.

### 2.3. Group Formation

Based on PPI intake (*n* = 363) and non-intake (*n* = 82) at half a year pTx, two patient groups were formed. These were used for a direct comparison of GFR and change thereof.

For the outcome measures >30% and >50% eGFR decline and the number of rejections, the groups 0 mg, 1–20 mg, 21–40 mg and >40 mg mean daily PPI intake were compared. The standard dose at our center is 40 mg pantoprazole, 20 mg is the common reduced dose, and above >40 mg (often 80 mg) is an elevated dose (rationale for the group formation).

### 2.4. Outcome Measures

Primary outcome measures were: the eGFR (at six months, one year, two years, three years and four years), change in the eGFR (from six months pTx to one year, two years, three years and four years), eGFR decline >30% and eGFR decline >50% (from six months to two years and two years to four years). All eGFR-values were calculated using the CKD-EPI (Chronic Kidney Disease Epidemiology Collaboration) equation [31].

Our secondary outcome was biopsy proven acute rejection (BPAR) in months one to six, seven to twelve and in the second year pTx. For each time period, every patient with a rejection was counted (not only a patient’s first rejection). The usual indication for biopsy in our center is a rise in creatinine with no apparent cause.

### 2.5. Statistical Analysis

Statistical analysis was performed using IBM SPSS^®^ Statistics 24 for Windows (IBM Corporation, Somers, NY, USA). Microsoft Excel was used for data collection, simple calculations, and graphing. 

This is an explorative study and no adjustment was made for multiple testing. *p*-values ≤ 0.05 were regarded as statistically noticeable. Normally distributed continuous variables are displayed as mean ± standard deviation (SD), non-normally distributed as median and interquartile range (IQR), and categorial variables as frequencies and percentage of total. Pairwise comparisons of independent samples were performed using student’s *t*-test or Welch’s unequal variance *t*-test for normally distributed data and the Mann–Whitney U test was used for non-normally distributed data. For categorial variables, groups were compared using Fisher’s exact test.

For the group comparison of the eGFR and change in the eGFR, the Mann–Whitney U test and multivariable linear regression were performed. Further information on model building and the included variables is found in Supplementary Materials 2A.

A possible relationship between >30% and >50% eGFR decline endpoints and mean daily PPI intake was investigated using Fischer’s exact test and multivariable logistic regression analysis. Mean daily PPI intake was calculated by averaging the prescribed PPI dose at half year intervals up to half a year before the final relevant eGFR value. Information on model building and the herein included variables can be found in Supplementary Materials 2B.

Correlation of BPAR in months one to six, seven to twelve and in the second year pTx with mean daily PPI intake, respectively, was investigated univariably using Fischer’s exact test and multivariably using logistic regression analysis with forward selection of confounders (same procedure as for >30% and >50% eGFR decline endpoints). A list of the variables included and further information on the testing can be found in Supplementary Materials 2C.

## 3. Results

### 3.1. Patients

A total of 511 patients were transplanted in the study period. The following exclusions were made: 37 patients as they were <18 years of age, 13 as they did not receive PPIs at primary discharge, five due to non-onset of graft function after transplantation and one due to death before primary discharge. A final number of 455 patients were included; the median follow-up time was 3.3 years (IQR, 2.2–4.9). In our cohort, 12 patients died and 10 experienced graft loss within the first year; seven died and eight graft losses occurred within the second year pTx. Most patients (96%) initially received pantoprazole as their PPI agent. Patient and donor characteristics are displayed in Table 1 (and Supplementary Materials 1).

Lost to follow-up rates in the PPI group and no PPI group were 16/363 (4.4%) and 2/82 (2.4%) at one year, 64/363 (17.6%) and 6/82 (7.3%) at two years, 152/363 (41.9%) and 28/82 (34.1%) at three years, 229/363 (63.1%) and 54/82 (65.9%) at four years, respectively.

### 3.2. Group Comparison

The two groups differed noticeably regarding recipient age, donor type (living or deceased), time dialyzed before transplantation, active smoking, smoking history, cerebral artery occlusive disease or stroke, coronary artery disease or myocardial infarction and Charlson index at the time of transplantation. These characteristics were favorable for the no PPI group. A noticeable difference was also found in the number of fast tacrolimus metabolizers three months pTx. (Table 1 and Supplementary Materials 1).

**Figure 1:**
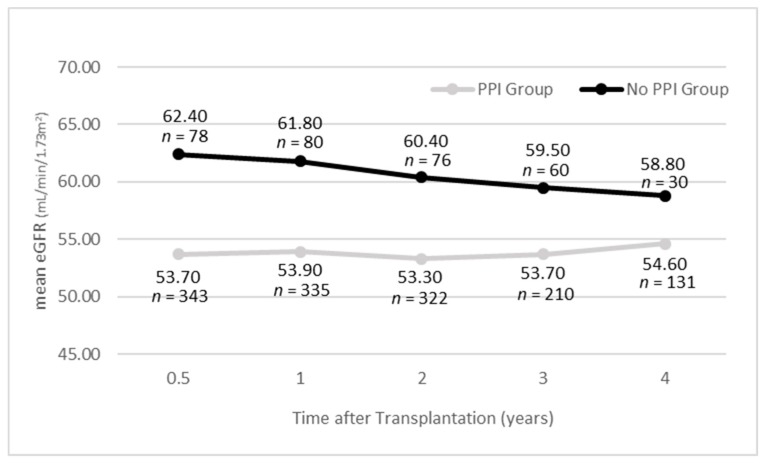
Trend in mean estimated glomerular filtration rate (eGFR). Mean eGFR is plotted against time in the two patient groups. Grouping is according to PPI intake or non-intake at half a year post-transplantation.

The PPI group showed significantly lower eGFR compared to the no PPI group at half a year, one year and two years pTx (*p* < 0.05) (multivariable). For the third and fourth year, the difference was not statistically significant (Supplementary Materials 3). The trend in the mean eGFR can be seen in Figure 1.

Regarding the change in the eGFR, both groups were similar over all time periods (Table 2).

### 3.3. >30% and >50% eGFR Decline

Multivariable logistic regression was only carried out for the outcome >30% eGFR decline from half a year to two years pTx. In all others, only univariable analysis was performed (due to the low number of events). eGFR decline >30% from half a year to two years showed statistical correlation with higher PPI doses in multivariable logistic regression (*p* = 0.044). All other eGFR decline endpoints showed no relation to mean daily PPI intake. eGFR decline >50% from two to four years showed some tendency in the same direction (*p* = 0.056). However, here, only three events occurred. Tables with the results can be viewed in Supplementary Materials 4A–D.

### 3.4. Secondary Outcomes

**Figure 2:**
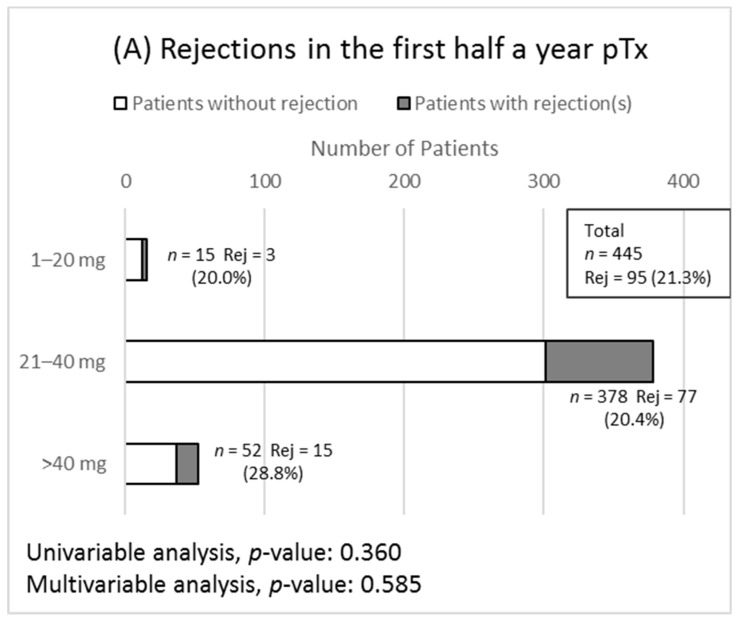

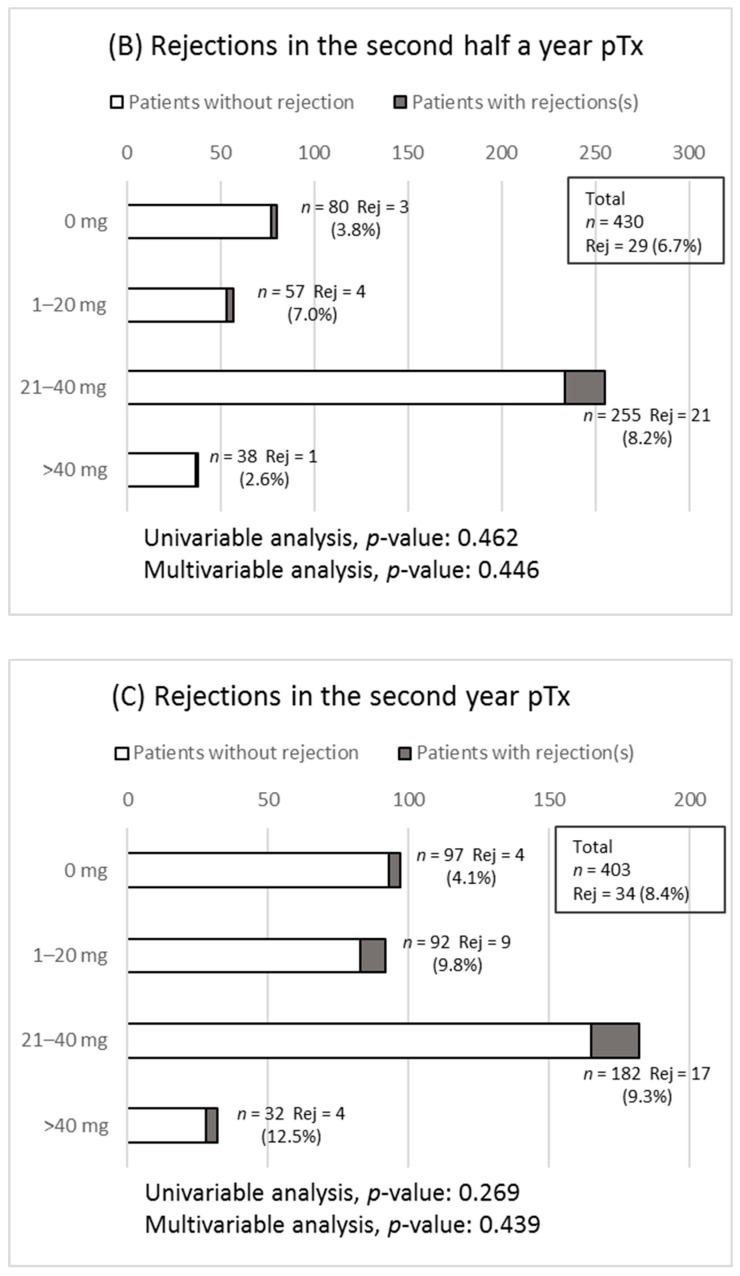
Results of the biopsy proven rejection analyses are shown. Grouping is according to PPI intake (pantoprazole equivalent) in the relevant time period. Rejection prevalence is shown as absolute number and as percentage of patients in the group. The dark part of each bar represents the number of patients with rejections. Abbreviations: *n*, total number of patients in the group; Rej, number of patients who experienced a graft rejection; pTx, post transplantation. (**A**) Analysis of rejections in months 1–6 pTx. (**B**) Analysis of rejections in months 7–12 pTx. (**C**) Analysis of rejections months 13–24 pTx.

BPAR occurred in 96 patients in months one to six, in 32 patients in months seven to twelve and in 36 patients in the second year pTx. For the rejection analysis, patients who did not complete a follow up of at least 5/6 of the analyzed time points were excluded from analysis. Those with death or transplant loss with prior rejection, however, were included. Twelve patients were excluded from multivariable analyses due to missing data. The results of the analysis are presented in Figure 2A–C. Regarding rejection types, mean daily PPI intake only showed a correlation with antibody mediated rejections (AMR) in the second year pTx in univariable analysis (*p* = 0.027); multivariable (logistic regression) analysis was not feasible in this case due to zero events in one group. All other tests did not show any association.

As the possible drug interaction of MMF with PPIs was of special interest, all rejection analyses were repeated, analyzing the data of patients who had MMF at primary discharge only. None of these tests showed any significant association.

## 4. Discussion

Our results demonstrate that prolonged PPI intake after KTx does not lead to any meaningful decline in kidney function within the first four years after transplantation. Additionally, our analyses of rejection rates are in line with recent studies showing no relevant association between rejection rates and PPI-intake after KTx [22,24,25].

Several large epidemiological studies observed a relationship between AKI, CKD, and PPI intake [1,2,3,4,5]. From case studies, an association between PPI intake and interstitial nephritis was previously assumed [32]. Estimates of the impact of this finding have not been around as long [33,34,35]. However, the relevance and the stake of interstitial nephritis for AKI in PPI observational studies still remains unclear [35,36]. As AKI can lead to CKD, it was not surprising that an association between CKD and PPI intake was recently proposed [1]. Nevertheless, Xie and colleagues provided evidence that PPI-associated CKD even occurred in the absence of AKI [3]. The pathomechanism, however, remains unknown. Proposed mechanisms include elevation of plasma asymmetric dimethylarginine levels [37], microinflammation due to gut microbe dysbiosis [38], endothelial senescence [39] and PPI-induced hypomagnesemia [40]. In addition to hypomagnesemia, PPI intake was recently associated to be dose-dependently linked to iron deficiency and hypomagnesemia in a kidney transplant cohort from the Netherlands [41,42]. The authors speculated that the effects were associated with reduced intestinal absorption of both elements under PPI therapy. However, our center’s policy is to monitor the iron status and to replace magnesium after KTx, because iron-deficiency is common and calcineurin inhibitors frequently lead to magnesium loss. Considering these findings, our results may relieve unwarranted fear when prescribing PPIs in KTx patients.

Following the Kidney Disease Improving Global Outcomes (KDIGO) guideline, a form of mycophenolate acid (MPA) together with the calcineurin inhibitor tacrolimus and low dose corticosteroid therapy is the preferred maintenance therapy after KTx at our center (Table 1 and Supplementary Materials 1) [43]. The tacrolimus target level in our center was 6–10 ng/mL from months one to three and 4–8 ng/mL for the following time.

Tacrolimus and PPI potentially interfere e.g., at the cytochrome P450 system (CYP3A) [17,18]. Another mechanism proposed is that PPI can increase the uptake of tacrolimus in the small intestine [19,44]. Both mechanisms could increase blood tacrolimus levels. Usually, these interactions are not clinically noticeable as several factors have a more profound effect on tacrolimus metabolism and exposure [45]. However, as tacrolimus can be nephrotoxic, slight increases in exposure may be relevant in the course of time [18,46]. Although analysis of tacrolimus blood levels was not the goal of this study, it is interesting to note that tacrolimus blood levels at three months pTx showed a tendency (*p* = 0.07) to be higher in the PPI group (Supplementary Materials 1). Furthermore, significantly more fast tacrolimus metabolizers were found in the no PPI group (*p* = 0.035). However, the previously mentioned Dutch KTx magnesium study found no relevant interaction between PPI and tacrolimus in their observational study [42]. Thus, these interesting findings may warrant further investigation.

It may also be worth mentioning that we did not investigate a possible effect of PPIs on the intrapatient variability of tacrolimus. It has been shown that the intrapatient variability of tacrolimus correlates with poor long-term outcomes in kidney transplant recipients [47]. It may be assumed that any drug with the possibility of interfering with tacrolimus pharmacokinetics may potentially change the intrapatient variability of tacrolimus [48]. To our knowledge, no study exists which has directly investigated a potential relationship between PPI intake and increased intrapatient variability in kidney transplant recipients. However, we suspect this effect to be minimal when adherence to our center’s instruction of ingesting tacrolimus on an empty stomach is followed. As we did not see any differences regarding the eGFR changes between the groups, a clinically relevant effect of PPIs on transplant function does not seem to exist.

Mycophenolate mofetil is a prodrug that is hydrolyzed to the active metabolite MPA. It acts as a selective uncompetitive inhibitor of inosine monophosphate dehydrogenase (IMPDH) and thus inhibits de novo guanosine synthesis [6]. Pharmacokinetic studies have shown that PPIs may reduce MPA exposure in patients receiving concomitant MMF and PPI treatment, whereas the alternative drug enteric-coated mycophenolate sodium is not affected [6,7,8,9,10,11,12]. Decreased MPA exposure can increase rejection rates [13,14,15,16]. However, in a recent pharmacokinetic blinded cross-over study [49], this mentioned interaction was not found. In line with this and with previously published observational studies, rejection rates in our cohort were comparable between groups [22,24,25].

Previous PPI studies in KTx patients relevant to our investigation have focused on the possible interaction of MMF with PPIs. Van Boeckel and colleagues compared 125 patients taking pantoprazole with 77 patients using ranitidine [22]. The primary outcome was BPAR and secondary outcomes were creatinine and the eGFR at three months pTx. No significant differences were found in any of the outcomes. Knorr and colleagues [24] came to similar results in their comparative study of 213 patients receiving PPIs, and 390 with ranitidine. The primary outcome was BPAR in the first year pTx. Both groups had comparable rejection rates and eGFRs. Notably, in the subgroup of African American recipients (predominantly fast tacrolimus metabolizers), PPI intake and rejection rates correlated. The recent study by Patel and colleagues [25] compared rejection rates in 183 patients taking PPIs and 339 using histamine-2 receptor antagonists. The primary outcome was the incidence of acute rejection within one year pTx, but eGFR values at one month and one year were also compared between the two groups. None of these parameters showed a significant difference.

Our study adds to the existing literature as we extended the analyses of the eGFR and changes thereof to a longer time period (half a year to four years) than previous studies (analyzing three to twelve months).

A simple comparison of the eGFR reflected differences in patient characteristics (Table 1 and Supplementary Materials 1). These differences were not a result of PPI therapy as is shown by comparable changes in the eGFR in both groups during follow-up (Table 2).

Moreover, similar rejection rates in the groups taking different doses of PPI, namely 0 mg, 1–20 mg, 21–40 mg and >40 mg pantoprazole equivalent, led us to conclude that the relevance of PPI intake for MMF efficacy is at best minimal. This is in line with the previously mentioned observational studies that used different methodologies. Correction for various potentially relevant confounders did not change the results with regards to kidney function or rejection rates. In line with our observations is a recently published meta-analysis that evaluated the data of 6786 KTx patients. The authors found that PPI use was linked to hypomagnesemia, but not associated with acute rejection, graft loss, or one-year mortality [40].

One limitation of our study is the retrospective study design analyzing a limited number of patients from one center. For part of the rejection analysis and >30% and >50% eGFR decline, the inclusion of covariables in the multivariable logistic regression analyses was limited due to the low number of events. Confounding by indication proved a serious difficulty in this study. Patients with longer, higher PPI intake showed a tendency to higher comorbidity and risk factors (Table 1 and Supplementary Material 1). To cope with this, we included information on the Charlson comorbidity index. PPI therapy post-transplant is the standard of care at our center. Discontinuance of PPI medication was assumedly due to clinical evaluation or patient choice, not by standard procedure.

For the statistically observed relation of second year AMRs and >30% eGFR decline (from half a year to two years) with PPI average intake, we presume residual confounding to be the reason because in a post-hoc comparison of the groups used for the >30% eGFR decline analysis, we found significant differences for the following patient characteristics: recipient age and BMI, pre-transplant dialysis time, prior renal transplant, donor type (living or deceased), pre-transplant diabetes, ABO transplant incompatibility, cerebral arterial occlusive disease or stroke, coronary heart disease or myocardial infarction, statins, prior smoking history, continuation of smoking after KTx, and Charlson comorbidity index. All of these, apart from prior renal transplant and ABO incompatibility, showed an unfavorable tendency with higher PPI intake.

## 5. Conclusions

We conclude from our data that prolonged PPI therapy is safe in regard to KTx function. However, further studies into a possible interaction between PPIs and tacrolimus may be of interest. In addition, our findings highlight the importance of examining changes in the eGFR rather than single eGFR measurements in similar studies. Polypharmacy is a relevant problem in the transplant population [50] and it is always advisable to question unnecessary medication [51]. This may include PPI therapy.

## Figures and Tables

**Table 1:** Patient characteristics at primary hospital discharge post-transplantation (additional information included in Supplementary Materials 1).

Patient Characteristic	All Patients (*n* = 455)	PPI Group (*n* = 363)	No PPI Group (*n* = 82)	*p*-Value of Group Comparison
Recipient age, mean ± SD (years)	52.6 ± 14.2	53.1 ± 13.9	49.3 ± 14.5	0.026
Recipient male gender, *n* (%)	279 (61.3)	219 (60.3)	52 (63.4)	0.707
Recipient BMI, mean ± SD (kg/m^2^)	25.9 ± 4.4	26.0 ± 4.3	24.9 ± 4.5	0.053
Prior renal transplantation, *n* (%)	64 (14.1)	45 (12.4)	16 (19.5)	0.109
Age of donor, mean ± SD (years)	53.1 ± 14.0	53.3 ± 14.4	51.4 ± 11.9	0.204
Living donor, *n* (%)	153 (33.6)	112 (30.9)	41 (50.0)	0.001
Male donor, *n* (%)	208 (45.7)	170 (46.8)	33 (40.2)	0.326
Delayed graft function, *n* (%)	79 (17.4)	59 (16.3)	11 (13.4)	0.616
European Senior Program, *n* (%)	76 (16.7)	62 (17.1)	10 (12.2)	0.322
Cold ischemia time (hours), median (IQR)	7.8 (2.5–11.6)	7.8 (2.7–11.7)	5.2 (2.3–11.1)	0.053
Pre-Tx time dialyzed (months), median (IQR)	45.3 (21.0–86.0)	48.2 (23.2–88.5)	32.4 (8.6–67.2)	0.002
Tacrolimus therapy at primary discharge, *n* (%)	432 (94.9)	347 (95.6)	76 (92.7)	0.265
Cyclosporin therapy at primary discharge, *n* (%)	23 (5.1)	16 (4.4)	6 (7.3)	0.265
MPS therapy at primary discharge, *n* (%)	76 (16.7)	57 (15.7)	18 (22.0)	0.191
MMF therapy at primary discharge, *n* (%)	341 (74.9)	278 (76.6)	57 (69.5)	0.200
MMF mean daily dosage (mg), median (IQR)	1000 (500–1000)	1000 (500–1000)	1000 (0–1063)	0.851
Cortisone intake at primary discharge, *n* (%)	444 (97.6)	353 (97.2)	81 (98.8)	0.698
CCI, median (IQR)	2 (2–4)	3 (2–4)	2 (2–3)	<0.001
HLA mismatch on A, B and DR, mean ± SD	2.9 ± 1.7	2.9 ± 1.7	2.9 ± 1.7	0.875
Basiliximab induction, *n* (%)	363 (79.8)	293 (80.7)	61 (74.4)	0.272
ATG induction, *n* (%)	14 (3.1)	13 (3.6)	1 (1.2)	0.482
ABO blood type incompatible transplant, *n* (%)	37 (8.1)	26 (7.2)	11 (13.4)	0.077
PRA >20%, *n* (%)	60 (13.2)	48 (13.2)	10 (12.2)	1.000

The two compared groups were formed based on PPI (proton pump inhibitor) intake (PPI Group) or non-intake (No PPI Group) at half a year post-transplantation. Results are presented as mean ± standard deviation (SD), median and interquartile range (IQR) or as absolute and relative frequencies. Abbreviations: BMI, body mass index; Tx, transplantation; MPS, enteric-coated mycophenolate sodium; MMF, mycophenolate mofetil; CCI, Charlson comorbidity index; HLA, human leukocyte antigen; ATG, Antithymocyte globulin; PRA, panel reactive antibodies. Along with HLA mismatch (*n* = 3) and Basiliximab induction (*n* = 7), four other variables have one patient with missing values.

**Table 2:** Results of the PPI group comparison of change in the eGFR.

Time Period of Analyzed eGFR Change	Groups 1 = PPI 0 = No PPI	*n*	Mean Change in the eGFR ± SD (mL/min/1.73 m^2^)	Median (IQR) Change in the eGFR	*p*-Value in Univariable Analysis	*p*-Value (CI) in Multivariable Linear Regression Model
0.5–1 year	1	323	−0.6 ± 12.0	1.0 (−6.0–6.5)	0.488	0.498 (−1.9–3.8)
	0	78	−0.5 ± 8.3	−0.5 (−5.6–4.4)		
0.5–2 years	1	310	−1.6 ± 14.2	0.2 (−9.0–7.5)	0.274	0.542 (−2.3–4.5)
	0	74	−2.4 ± 10.2	−1.5 (−9.2–5.2)		
0.5–3 years	1	202	−0.8 ± 15.1	0.35 (−8.0–8.4)	0.331	0.452 (−2.6–5.8)
	0	58	−2.5 ± 11.0	−1.3 (−7.0–5.4)		
0.5–4 years	1	125	−0.1 ± 14.5	−0.1 (−7.3–8.6)	0.101	0.228 (−2.2–9.1)
	0	28	−4.2 ± 9.1	−1.7 (−10.5–2.9)		

Results of the comparison of the change in the eGFR, values between groups. The eGFR value measured at half a year is always used as the reference value and was subtracted from that of the later date. Groups were formed based on PPI intake or non-intake at half a year pTx (post-transplantation). For the linear regression models, the patient number is slightly reduced (<3 patients difference per test) due to missing covariables in a few patients. Abbreviation: CI, confidence interval.

## Supplementary Materials

The following are available online at https://www.mdpi.com/2077-0383/9/1/258/s1. Supplementary Materials 1: Additional patient information; Supplementary Materials 2: Statistical analysis; Supplementary Materials 3: Results of the group comparison of the absolute eGFR; Supplementary Materials 4: Results of the >30% and >50% eGFR decline with mean daily PP-dose tests.
