# Supplementary material for: The Effect of Proton Pump Inhibitor Use on Renal Function in Kidney Transplanted Patients
Source: J Clin Med. 2020 Jan 18;9(1):258. doi: 10.3390/jcm9010258 (PMC7019820; doi:10.3390/jcm9010258)
Supplement: Supplementary file 1 [file jcm-09-00258-s001.pdf]

Supplementary Materials 1 (Additional patient information).

Patient Characteristic	All Patients (n=455)	PPI-Group (n=363)	No PPI-Group (n=82)	p-value
Pre-transplant arterial hypertension, n (%)	434 (95.4)	348 (95.9)	76 (92.7)	0.246
CAOD or stroke, n (%)	106 (23.3)	92 (25.3)	11 (13.4)	0.020
CAD or myocardial infarction, n (%)	93 (20.4)	84 (23.1)	6 (7.3)	0.001
Active smoker pTx, n (%)	53 (11.6)	48 (13.2)	4 (4.9)	0.035
Former smoker, n (%)	121 (26.6)	106 (29.2)	13 (15.9)	0.013
Pre-transplant diabetes, n (%)	70 (15.4)	54 (14.9)	10 (12.2)	0.605
PAOD, n (%)	87 (19.1)	71 (19.6)	12 (14.6)	0.349
Anticoagulants, n (%)	36 (7.9)	30 (8.3)	4 (4.9)	0.364
Antiplatelet drugs, n (%)	121 (26.6)	101 (27.8)	16 (19.5)	0.129
Statin, n (%)	169 (37.1)	142 (39.1)	24 (29.3)	0.102
Pre-transplant DSA, n (%)	16 (6.6)	13 (6.7)	3 (5.9)	1.000
Cortisone intake one-year pTx, n (%)	402 (88.4)	328 (90.4)	73 (89.0)	0.406
Tacrolimus dosage 3 months pTx (mg), median (IQR)	6.0 (4.0-8.0)	5.5 (4.0-8.0)	6.0 (3.5-8.0)	0.960
Tacrolimus blood levels 3 months pTx, mean±SD	7.8±2.5	7.9±2.5	7.2±2.2	0.070
Fast tacrolimus metabolizer (3 months pTx), n (%)	125 (35.5)	96 (33)	29 (47.5)	0.039

The two compared groups were formed based on PPI-intake or non-intake at half a year post-transplantation. Results are presented as mean ± standard deviation (SD), median and interquartile range (IQR) or as absolute and relative frequencies. Abbreviations: pTx, post-transplantation; CAOD, cerebral artery occlusive disease; CAD, coronary artery disease PAOD, peripheral arterial occlusive disease; DSA, donor specific antibodies. Fast tacrolimus metabolizers were defined as blood tacrolimus concentration (ng/ml)/dose (mg) < 1.05. 205 patients had no information on pre-transplant DSA, 30 for cortisone intake one-year pTx, in 101 patients information was not available for tacrolimus metabolism speed and for 100 patients tacrolimus blood levels at 3 months pTx was missing (largely due to different medication). For these three variables, the percentages shown are only in regard of those patients with available information. Besides these, only CAOD had one missing value.

Supplementary Materials 2 (A–C) (Statistical analysis).

(A) Supplemental information on Model building (multivariable linear regression) in the group comparison of the eGFR and change in the eGFR: For multivariable analysis, model building was carried out taking the half-year grouping in a first block and confounders in a second block with forward selection (inclusion: score-test p-value <0.5, exclusion: likelihood-ratio test p-value ≥0.10). The following confounders were included: recipient and donor age and sex; recipient BMI; completion of prior renal transplants; transplant under European Senior Program; donor type (living or deceased); DGF; cold ischemic time; pre-transplant time dialyzed; pre-transplant arterial hypertension; pre-transplant diabetes; presence of peripheral arterial occlusive disease; cerebral arterial occlusive disease or stroke; coronary heart disease or myocardial infarction; anticoagulant medication; antiplatelet drugs; statins; MMF in-take at primary discharge (categorial); prior smoking history; continuation of smoking after KTx; and Charlson comorbidity index at transplantation.

(B) Supplemental information for Model building with the >30% and >50% eGFR-decline endpoints: For multivariable logistic regression, model building was carried out taking the grouped mean daily PPI-intake in a first block and confounders in a second block with forward selection (inclusion: score-test p-value <0.5, exclusion: likelihood-ratio test p-value ≥0.10). The same confounders were used as for the group comparison of eGFR and change in eGFR.

(C) Supplemental information for the testing of BPAR with mean daily PPI-intake: The following variables stood for selection in model building: recipient and donor age and sex; completion of prior renal transplants; MMF mean dosage (calculated by averaging the MMF dosage pTx and that in the second year pTx); basiliximab induction therapy; anti-thymocyte globulin induction therapy; intake of cortisone at primary discharge (for analyses up to one year) or intake of cortisone at one year pTx (for analyses of the second year); DGF; number of HLA mismatches; ABO incompatibility; donor type; cold ischemia time; panel reactive antibodies (PRA) over 20%; DSA before transplantation and tacrolimus blood level three months pTx. T-cell mediated rejections (TCMR) and antibody mediated rejections (AMR) were also analyzed separately for each time period. As routine pre transplant DSA testing only started in September 2012, this data was not available for 205 patients. For tacrolimus blood level three months pTx, 100 patients had missing information. In order to allow for a maximum number of patients but also not disregard a potential effect of important confounders, we repeated all analyses first excluding, then including both variables. This did not change the overall result of any test (with respect to the level of significance).

Supplementary Materials 3 (Results of the group comparison of the absolute eGFR).

Point in time (post-transplant)	Group 1= PPI 0= No PPI	n	Mean± SD (mL/min/1.73 m2)	Median (IQR)	p-value in Univariable analysis	p-value (CI) in multivariable linear regression model
½-Year eGFR	1	343	53.7 ± 20.9	52.1 (38.4 – 68.3)	<0.001	0.009 (-10.5 – -1.5)
	0	78	62.4 ± 19.5	61.4 (48.0 – 74.5)		
1-Year eGFR	1	335	53.9 ± 19.9	52.0 (39.9 – 67.3)	0.001	0.009 (-9.8 – -1.4)
	0	80	61.8 ± 19.7	61.6 (45.9 – 75.1)		
2-Year eGFR	1	322	53.3 ± 20.0	51.0 (40.4 – 65.0)	0.003	0.031 (-9.1 – -0.4)
	0	76	60.4 ± 19.4	61.8 (45.4 – 73.5)		
3-Year eGFR	1	210	53.7 ± 21.5	51.0 (38.6 – 68.0)	0.039	0.186 (-9.1 – 1.8)
	0	60	59.5 ± 21.1	59.7 (43.8 – 78.2)		
4-Year eGFR	1	131	54.6 ± 20.6	52.0 (40.2 – 67.6)	0.272	0.834 (-8.4 – 6.8)
	0	30	58.8 ± 17.7	61.4 (44.7 – 73.0)		

Groups were formed based on PPI-intake or non-intake at half a year pTx. For the multivariable linear regression models, the patient number is slightly reduced (<3 patients difference per test) due to missing covariables in a few patients. Abbreviations: SD, standard deviation; PPI, proton pump inhibitor.

Supplementary Materials 4 (A-D) (Results of the >30% and >50% eGFR decline with mean daily PP-dose tests).

Supplementary Materials 4 (A) Over 30% eGFR decline ½ year to 2 years pTx.

Mean daily PPI-dose	n	No. of Pat. >30% decline (%)
0 mg	56	3 (5.4)
1-20 mg	89	11 (12.4)
21-40 mg	203	17 (8.4)
>40 mg	36	8 (22.2)
Total	384	39 (10.2)

Univariable analysis, p-value: 0.052. Multivariable logistic regression analysis, p-value: 0.044.

Supplementary Materials 4 (B) Over 50% eGFR decline ½ year to 2 years pTx.

Mean daily PPI-dose	n	No. of Pat. >50% decline (%)
0 mg	56	1 (1.8)
1-20 mg	89	5 (5.6)
21-40 mg	203	5 (2.5)
>40 mg	36	1 (2.8)
Total	384	12 (3.1)

Univariable analysis, p-value: 0.511.

Supplementary Materials 4 (C) Over 30% eGFR decline 2 years to 4 years pTx.

Mean daily PPI-dose	n	No. of Pat. >30% decline (%)
0 mg	48	1 (2.1)
1-20 mg	37	2 (5.4)
21-40 mg	55	4 (7.3)
>40 mg	21	2 (9.5)
Total	161	9 (5.6)

Univariable analysis, p-value: 0.501.

Supplementary Materials 4 (D) Over 50% eGFR decline 2 years to 4 years pTx.

Mean daily PPI-dose	n	No. of Pat. >50% decline (%)
0 mg	48	0 (0)
1-20 mg	37	0 (0)
21-40 mg	55	1 (1.8)
>40 mg	21	2 (9.5)
Total	161	3 (1.9)

Univariable analysis, p-value: 0.056.

Results of the 30 and 50% eGFR-decline analyses. Each parameter and time period are shown in separate tables. Abbreviations: pTx, post transplantation.
